# Chitin-Derived Nanocatalysts for Reductive Amination Reactions

**DOI:** 10.3390/ma16020575

**Published:** 2023-01-06

**Authors:** Daniele Polidoro, Daily Rodriguez-Padron, Alvise Perosa, Rafael Luque, Maurizio Selva

**Affiliations:** 1 Department of Molecular Science and Nanosystems, Ca’ Foscari University of Venice, Via Torino 155, 30175 Venezia Mestre, Italy; 2 Department of Natural Sciences, Mid Sweden University, Holmgatan 10, 85170 Sundsvall, Sweden; 3 Universidad ECOTEC, Km. 13.5 Samborondón, Samborondón EC092302, Ecuador

**Keywords:** biomass valorization, chitin-derived carbonaceous supports, supported nanoparticles, reductive amination, furfural

## Abstract

Chitin, the second most abundant biopolymer in the planet after cellulose, represents a renewable carbon and nitrogen source. A thrilling opportunity for the valorization of chitin is focused on the preparation of biomass-derived *N*-doped carbonaceous materials. In this contribution, chitin-derived *N*-doped carbons were successfully prepared and functionalized with palladium metal nanoparticles. The physicochemical properties of these nanocomposites were investigated following a multi-technique strategy and their catalytic activity in reductive amination reactions was explored. In particular, a biomass-derived platform molecule, namely furfural, was upgraded to valuable bi-cyclic compounds under continuous flow conditions.

## 1. Introduction

Due to the current demand to find alternatives for the replacement of fossil resources, the attention of the scientific community has moved towards biomass as a renewable carbon source [1,2,3,4,5]. Biomass valorization could offer a myriad of possibilities to avoid a crisis in the supply of chemicals, materials and fuels, such as via the extraction of valuable compounds, the upgrading of biomass-derived platform molecules into high-added value chemicals or the direct utilization of biomass residues for the preparation of advanced materials [6,7,8].

The utilization of lignocellulosic biomass residues, typically composed of cellulose, hemicellulose and lignin, is a thrilling possibility, providing access to a wide range of platform chemicals. In particular, furfural, which is derived from cellulose and hemicellulose, represents an interesting platform chemical which could be employed in the synthesis of several compounds including levulinates, (tetrahydro)furfuryl alcohol, 2-methyl(tetrahydro)furan, cyclopentanone(l) and lactones, among others [9]. Moreover, a thrilling possibility for the upgrading of furfural is based on reductive amination reactions, which has been a topic recently investigated by our research group [10,11]. Such a chemical transformation could lead to the formation of various aminated products, which could find applications in different areas, such as pharmaceutical products, agrochemicals or corrosion inhibitors, just to name a few [12].

For the reductive amination of furfural, as well as other reactions for the upgrading of biomass-derived platform molecules, green chemistry principles need to be considered; looking forward, the development of sustainable processes, and hence the use of catalytic steps, becomes a necessity [8,13]. Therefore, the development of efficient, sustainable and easily recoverable catalytic materials has become a key concern for the chemical industry [14,15,16]. In this sense, the intelligent design of heterogeneous catalysts needs to consider several aspects related to the physicochemical, electronic and optical features of the materials [17]. Furthermore, the synthetic approach and the feedstocks employed in the preparation of the catalytic samples are critical factors to evaluate the sustainability of the overall process [18].

The use of biomass waste for the preparation of catalytic materials is a remarkable strategy, either employing biomass residues as a sacrificial template to control the textural properties of the samples or as a carbon source which could serve as support for the immobilization of, for instance, metal nanoparticles. Furthermore, the design of heteroatom-doped carbonaceous materials is also an interesting opportunity which has taken advantage of biomass waste composition for the low-cost synthesis of advanced materials [19,20]. In this regard, *N*-doped carbons have emerged as good candidates for the development of metal-free catalytic processes—where nitrogen sites could play a critical role due to their basic properties—or for employment as supports for metal/metal oxide nanoparticles. In the latter case, the presence of nitrogen could both favor the good dispersion of metal entities and ensure the stability of the catalytic samples, serving as anchor sites to avoid metal leaching [21,22,23,24].

Considering the aforementioned premises, herein we propose the preparation of palladium nanoparticles supported on *N*-doped carbons (Pd-N/C) employing chitin (the second most abundant biopolymer in the planet after cellulose) as a carbon and nitrogen source. Two synthetic strategies will be evaluated and compared for the preparation of the Pd-N/C materials, and their catalytic activity will be examined in the reductive amination reaction of furfural. Particularly, furfural reductive amination with furfuryl amine will be explored under continuous flow conditions, giving insights into the efficiency and stability of the designed samples.

## 2. Materials and Methods

### 2.1. Material and Equipment

Furfural, furfuryl amine, methanol, 2-propanol, chitin, EDTA and Pd(Ac)_2_ were commercially available compounds sourced from Sigma-Aldrich. If not otherwise specified, reagents and solvents were employed without further purification. Gas chromatography–mass spectrometry (GC-MS) (electron ionization (EI), 70 eV) analyses were performed on a HP5-MS capillary column (L = 30 m, Ø = 0.32 mm, film = 0.25 mm). GC (flame ionization detector (FID)) analyses were performed with an Elite-624 capillary column (L = 30 m, Ø = 0.32 mm, film = 1.8 mm). All reactions were performed in duplicate to verify reproducibility.

### 2.2. Synthesis of Supported Palladium Nanoparticles on Chitin-Derived N-Doped Carbonaceous Materials

Two synthetic procedures were employed, labeled as procedure A and B. Procedure A was based on the impregnation and aging of chitin (5 g) with a Pd(Ac)_2_ (0.5 mmol) solution in 2-propanol (15 mL). Subsequently, the material was oven dried at 100 °C under vacuum overnight and further calcined at 500 °C (heating rate was 5 °C/min) for 1 h under N_2_ flow (10 mL min^−1^) for 1 h. The catalytic sample achieved by this procedure was labeled as Pd-N/C_a_. For procedure B, Pd(Ac)_2_ (0.5 mmol) was dissolved in 2-propanol (60 mL), together with EDTA (1 g). Subsequently, chitin (5 g) was added to the mixture, which was kept under stirring for 9 h at 80 °C under reflux. The suspension was filtered and the so-obtained solid was dried at 100 °C overnight and, finally, heated at 500 °C for 1 h, according to the conditions previously described. The catalytic sample achieved by this procedure was labelled as Pd-N/C_b_. The resulting materials were ground to powder (particle size <200 μm) and stored in the oven (70 °C, 15 mbar) until further use. The yield of the obtained materials was ca. 25 ± 5%, based on the total weight of chitin and the metal precursor used.

### 2.3. Material Characterization

The crystalline structure of the samples was investigated by X-ray diffraction (XRD) in a D8 Advance diffractometer from Bruker^®^ AXS, using the X-ray source of the Cu Kα radiation, coupled to a LynxEye detector, and monitoring the 2θ within 8–80° at a rate of 0.08° min^−1^.

The textural properties, i.e., surface area, the volume of the pore and pore size, were determined from N_2_ physisorption at 77 K, performed in an ASAP 2000 instrument from Micromeritics^®^. The samples were outgassed at 120 °C for 2 h. Then, adsorption–desorption isotherms were recorded at −196 °C. The specific surface areas were calculated by the BET method; the pore volumes were calculated from adsorption isotherms and the pore size distributions were estimated using the Barrett, Joyner and Halenda (BJH) algorithm available as built-in software from Micromeritics (Micromeritics Instrument Corporation (Norcross, GA, USA)).

SEM-EDX images were acquired in a JEOL-SEM JSM-7800 LV scanning microscope. Transmission electron microscopy (TEM) was performed to observe the size and shape of the particles in a JEOL 2010 operated at an acceleration voltage of 200 kV and with a FEI Tecnai G2 system, equipped with a charge-coupling device (CCD) camera. Samples were ground and then suspended in ethanol, followed by dipping of a holey-carbon coated copper grid of 300 mesh which was left to dry under air for a few minutes prior to recording. ICP-OES analysis was carried out in the Avio 550 Max ICP-OES Optical Emission Spectrometer

### 2.4. Typical CF-Reductive Amination Reaction and Products Analysis

The catalytic performance of the catalysts was evaluated under liquid phase continuous flow conditions in an H-Cube Mini Plus™ flow hydrogenation reactor. In a typical continuous flow (CF) reductive amination of furfural, 1 equivalent of furfuryl amine (2) was added to a 0.1 M solution of furfural (1) in methanol. The mixture was kept under magnetic stirring for 5 min, affording the corresponding imine 3 which was pumped through at the desired temperature, hydrogen pressure and flow. Reactions were performed for 90 min, collecting samples every 15 min for further analysis. Samples collected at “0 min” were considered as first outcomes under operative reaction conditions passed through the cartridge. Conversion and selectivity were determined by GC-FID and the product structures were assigned by GC-MS. Characterization data are available in the SI Section, Figures S1–S4.

## 3. Results and Discussion

### 3.1. Synthetic Approach

**Figure 1:**
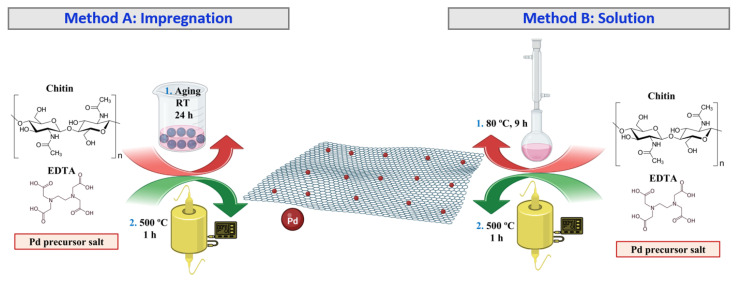
Schematic representation of the synthetic strategies considered for the preparation of Pd-N/C materials.

In this work, a series of palladium nanoparticles supported on biomass-derived *N*-doped carbons were prepared following two different strategies (Figure 1).

In one case (method A), chitin was impregnated and aged at room temperature with a palladium precursor solution, namely palladium acetate dissolved in a minimum amount of 2-propanol, while in the other case (method B), chitin was dispersed in a solution of palladium acetate with a proper amount of 2-propanol (namely 70 mL) and kept at 80 °C under stirring for 9 h. The impregnation method (A) possesses several advantages, especially from an environmentally friendly point of view, considering the minimum amount of solvents involved. However, the aforementioned protocol also has some drawbacks, such as the lack of homogeneity and the low control of the particle size and dispersion. On the other hand, the second protocol employed in this work for the synthesis of nanoparticles is based in a deposition-precipitation strategy, which could give access to a better control of the morphology, dimensions and dispersion of the synthesized nanoparticles in comparison with the impregnation strategy. In the following sections, the results achieved by employing each synthetic method will be discussed and compared. Furthermore, as briefly described in the introduction section, *N*-doped carbon systems were selected as support materials considering recent investigations demonstrating that metal–nitrogen interactions favor the incorporation and further stability of the metal entities. Furthermore, to ensure the good dispersion of the prepared nanoparticles, EDTA was used as a robust metal complexing agent. Chitin was selected as a sustainable source of both nitrogen and carbon. Along with its high abundance, it should be highlighted that finding such applications for chitin is a desirable goal, which in several fields has been hindered by its low solubility. Herein, we provide a possible strategy for chitin utilization, which could be further extrapolated to the utilization of chitin-containing biomass residues, such as crustacean exoskeleton wastes.

### 3.2. Materials Characterization

**Figure 2:**
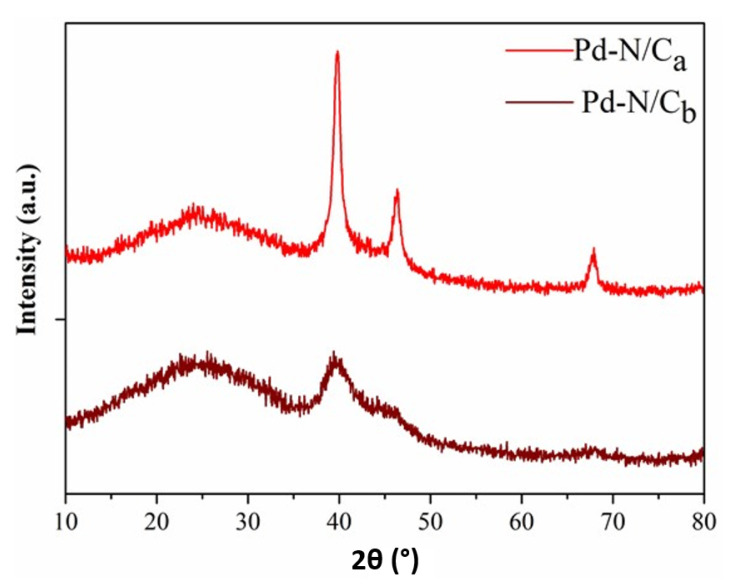
XRD pattern of the prepared Pd-N/C materials.

The crystal structure and arrangement of the prepared materials was evaluated by XRD analysis, as shown in Figure 2. Interestingly, clear differences could be found within both XRD patterns. As is displayed in Figure 2, the presence of a broad band around 24.0° could be observed for both materials, associated with the presence of amorphous carbon, in particular with the (002) crystallographic plane related to the parallel stacking of graphene-like sheets [25]. Moreover, the Pd-N/C_a_ sample showed the presence of sharp and well-defined signals located at 39.9°, 46.5° and 67.9°, which could be assigned to the (111), (200) and (220) crystallographic planes, respectively, of Pd(0) with a face-centered cubic crystal structure [26,27]. In addition, the XRD pattern of the Pd-N/C_b_ material revealed the presence of wider peaks, suggesting the nanosized nature of the palladium entities obtained by employing synthetic method B, most likely with the formation of smaller nanoparticles in comparison with those achieved by method A. In particular, for the Pd-N/C_b_ sample, the signal located at 39.8° was also attributed to the (111) crystallographic plane of metallic Pd, while the shoulder band at ca. 45.7° was correlated with the (200) plane of Pd (0). Such results were expected considering the nature of the employed synthetic approaches; in any case, microscopic analyses were further performed and are described in the following paragraphs.

**Figure 3:**
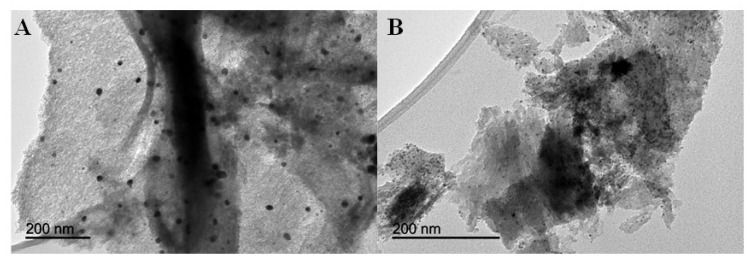
TEM images of the Pd-N/C_a_ (**A**) and Pd-N/C_b_ (**B**) samples.

**Figure 4:**
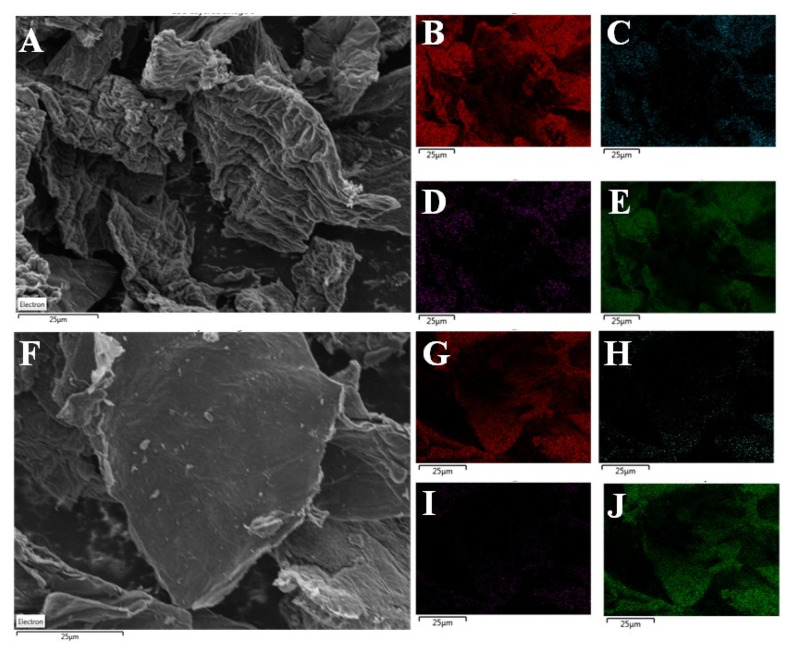
SEM-mapping micrographs of Pd-N/C_a_ and Pd-N/C_b_ materials. SEM images of Pd-N/C_a_ (**A**) and Pd-N/C_b_ (**F**). SEM-mapping results of Pd-N/C_a_ for carbon (**B**), nitrogen (**C**), oxygen (**D**) and palladium (**E**). SEM-mapping results of Pd-N/C_b_ for carbon (**G**), nitrogen (**H**), oxygen (**I**) and palladium (**J**).

The morphology of the catalytic materials was investigated by TEM analysis. TEM micrographs are displayed in Figure 3. In both the cases of Pd-N/C_a_ (Figure 3A) and Pd-N/C_b_ (Figure 3B), the formation of highly homogeneous and well-dispersed palladium nanoparticles, supported on a *N*-doped carbon matrix with a laminar structure, was found. Interestingly, the mean diameter of the obtained nanoparticles was found to be clearly smaller in the case of the sample prepared by procedure B. Specifically, the Pd-N/C_a_ displayed a mean radius of (18.5 ± 1.0) nm, while the Pd-N/C_b_ showed a mean radius of (1.5 ± 1.0) nm. These results were in accordance with the findings obtained by XRD analysis and confirmed the crucial influence of the synthetic approach on the final morphology and structure of the prepared samples. Moreover, the morphology and elemental composition of the samples were further evaluated by SEM-mapping analysis, as shown in Figure 4, confirming the successful incorporation of palladium entities on the *N*-doped carbon matrix. SEM-mapping micrographs revealed the presence of carbon, nitrogen, oxygen and palladium in both catalytic samples, and in all cases the elements were homogeneously distributed on the material.

Additionally, the textural properties of the samples were investigated by N_2_ physisorption measurements. According to the results reported in Table 1, in particular considering the mean pore size diameter (4.3 nm and 4.0 nm for Pd-N/C_a_ and Pd-N/C_b_, respectively), the prepared samples could be classified as mesoporous materials [17,28]. Both synthetic methodologies gave rise to the formation of mesoporous materials with similar mean pore size diameter and surface area values (300 m^2^/g and 311 m^2^/g for Pd-N/C_a_ and Pd-N/C_b_, respectively), as well as similar pore volume values (0.25 m^3^/g and 0.28 m^3^/g for Pd-N/C_a_ and Pd-N/C_b_, respectively). For comparison, chitin was treated following similar calcination conditions and the textural properties of the obtained *N*-doped carbon are also reported in Table 1. The results indicated that the textural features of the samples were mainly directed by the carbon–nitrogen matrix and by the calcination protocol, while neither the metal entities nor the strategies employed for their incorporation critically affected such properties. In any case, the slight decrease of surface area, increase of mean pore size diameter and decrease of pore volume of the palladium modified samples in comparison with the *N*-doped carbon support most likely indicated that the incorporation of metal entities led to the occlusion of smaller pores in the carbonaceous matrix. It is worth highlighting that the materials retained excellent textural properties, which could further favor their catalytic performance as will be described in the following sections. Pd concentrations determined by both ICP-OES and SEM-EDX are presented in Table 1. A comparison of the results indicated that the Pd component showed a good dispersion on the surface of the *N*-doped carbonaceous support, with a minimal contribution inside the pores of the sample. In fact, the superficial Pd concentration of both samples indicated a similar dispersion of the Pd component on the support, regardless of the synthetic approach.

### 3.3. Catalytic Activity

**Figure 5:**
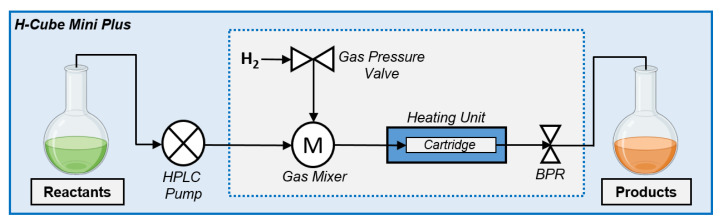
Schematic diagram of the H-Cube^®^ Mini Plus system.

To investigate the catalytic performance of the synthesized catalytic materials, Pd-N/C_a_ and Pd-N/C_b_, as well as commercial Pd/C, experiments were carried out in an H-Cube^®^ Mini Plus flow hydrogenation reactor (Figure 5). Catalytic materials were packed in 30 mm long ThalesNano CatCarts^®^ (around 50 mg per cartridge). The reactants solution was delivered by a high-performance liquid chromatography (HPLC) pump and conveyed to the continuous flow reactor placed inside a heating unit for temperature control. The required hydrogen pressure was generated in situ during the reaction by water electrolysis in the H-Cube equipment and mixed with the liquid phase by a gas mixer. A back-pressure regulator (BPR) maintained a constant operating pressure throughout the system and allowed for the depressurization and recovery of the reaction mixture. The recovered samples were directly analysed by GC to determine the reaction conversion and selectivity while the product structures were assigned by GC-MS. Characterization data are available in the SI Section, Figures S1–S4. All the reported reactions were run in duplicate to ensure reproducibility: unless otherwise stated, conversions and selectivity differed by less than 5% from one test to another.

**Scheme 1:**
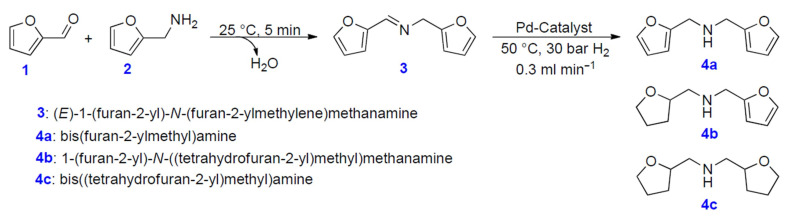
Reaction pathway in the reductive amination of furfural and furfuryl amine.

In this investigation, furfural was chosen as the model substrate to investigate the catalytic performance of the above-mentioned catalytic systems in reductive amination reactions. The feeding reactants solution was prepared by the addition of 1 equivalent of furfuryl amine (2) to a 0.1 M solution of furfural (1) in methanol (Scheme 1).

**Figure 6:**
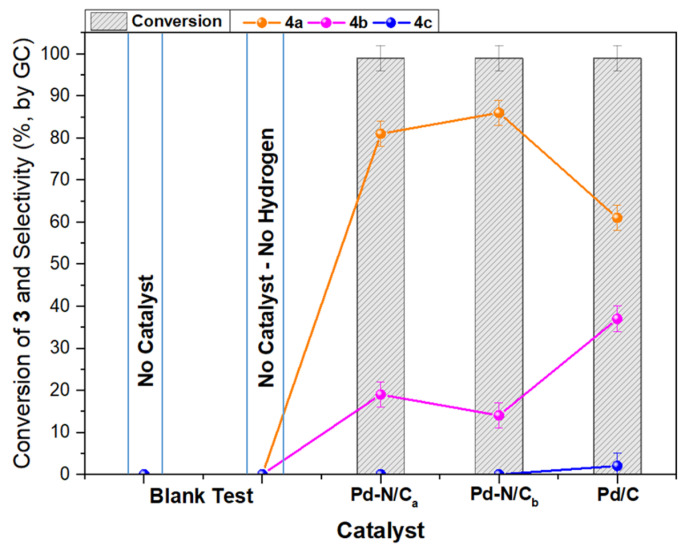
Catalysts screening in the reductive amination of furfural. Reaction conditions: 3 (0.1 M in methanol), catalyst (50 mg), 50 °C, 30 bar H_2_, flow rate (0.3 mL min^−1^). Conversion and selectivity were determined by GC.

The mixture was kept under magnetic stirring for 5 min, affording the corresponding imine 3. The resulting solution was delivered at 0.3 mL min^−1^ through the catalyst placed in the H-Cube^®^ Mini Plus reactor, under 30 bar H_2_ at 50 °C. The conversion of 3 and the selectivity towards the hydrogenation products 4a, 4b and 4c are summarized in Figure 6.

In order to confirm the essential role of the catalytic system in the reaction progress, blank measurements were performed in the absence of any catalyst. Furthermore, considering that hydrogen-donor solvents (especially methanol, ethanol and isopropanol) are often used to supply molecular hydrogen in hydrogenation reactions [29], blank tests were also performed in the absence of hydrogen pressure. This analysis revealed that in the absence of any catalyst and hydrogen pressure, the reaction displayed <1% conversion, confirming not only the fundamental role of the catalyst, but also that hydrogen gas was the only source. On the other hand, Pd-N/C_a_ and Pd-N/C_b_ showed comparable catalytic activity. Indeed, over both catalytic systems, quantitative conversion of 3 was achieved. However, a slight difference in the products’ distribution was observed. In detail, over Pd-N/C_a_, 4a and 4b were obtained with 81% and 19% selectivity, respectively, while over Pd-N/C_b_ the same products were obtained with 86% and 14% selectivity, respectively, suggesting the better performance of Pd-N/C_a_. Similar behavior was noticed when using commercial Pd/C. The conversion was stable and quantitative, but it brought about a change in the products’ distribution due to the concurrent formation of 4a (58%), 4b (36%) and the fully hydrogenated compound 4c (6%). Overall, considering the results reported in Figure 6, and the behavior of Pd-N/C_a_, this system was selected to continue the study through the investigation of the influence of major reaction parameters (temperature and pressure) in order to optimize the reaction conditions in terms of selectivity toward 4a and 4c.

#### Influence of Temperature and Pressure

**Figure 7:**
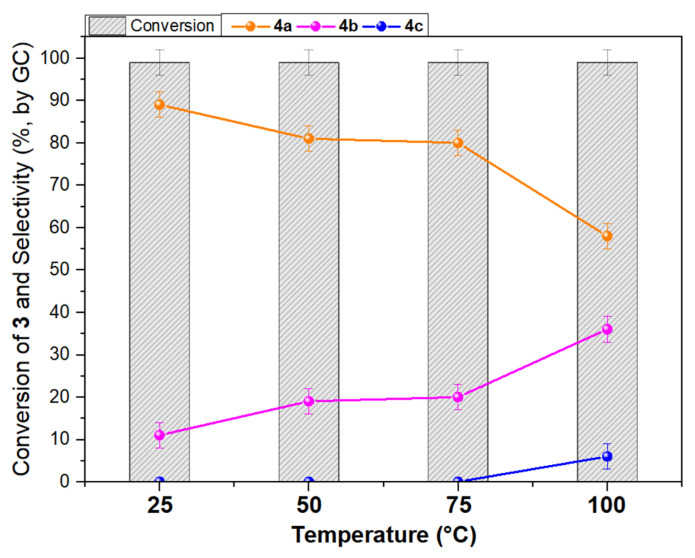
Effect of temperature in the reductive amination of furfural. Reaction conditions: 3 (0.1 M in methanol), catalyst (50 mg), 30 bar H_2_, flow rate (0.3 mL min^−1^). Conversion and selectivity were determined by GC.

The effect of the reaction temperature (T) was explored by performing a series of tests during which the reductive amination of furfural was carried out, changing T from 25 to 100 °C, at 0.3 mL min^−1^ under 30 bar H_2_ (the upper value of 100 °C was forced due to operative limitations of the equipment). The results are reported in Figure 7. Conversion and selectivity were determined by GC and GC/MS analyses of the reaction mixture confirmed the formation of the products of Scheme 1.

**Figure 8:**
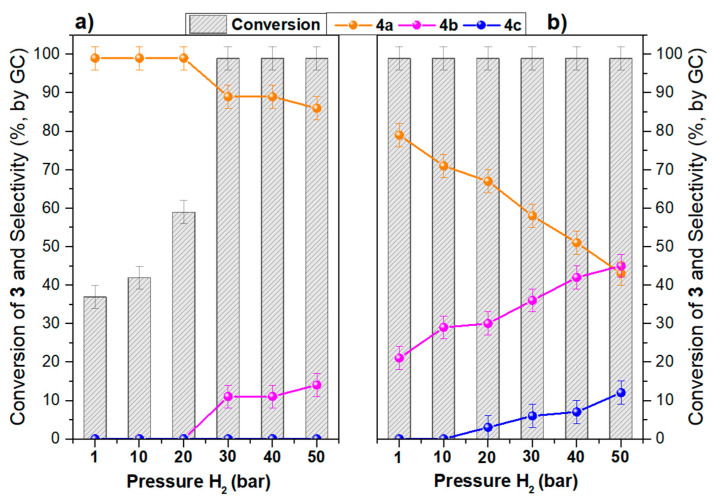
Effect of pressure in the reductive amination of furfural at (**a**) 25 °C and (**b**) 100 °C. Reaction conditions: 3 (0.1 M in methanol), Pd-N/C_a_ (50 mg), flow rate (0.3 mL min^−1^). Conversion and selectivity were determined by GC.

Interestingly, the full conversion of 3 was also observed at 25 °C, while a gradual increase in the reaction temperature drastically influenced the reaction outcome and the related products’ distribution. In this regard, by increasing the temperature from 25 to 100 °C, selectivity towards 4a decreased from 89% to 58%, respectively, implying the increase of 4b from 11% to 36%, respectively. Nevertheless, no noticeable changes in the formation of 4c, which increased to a maximum of 6% at 100 °C, were observed. With this result in hand, we were prompted to further optimize the reaction progress exploring the effect of the hydrogen pressure. To do that, further series of tests were carried out at 25 °C and 100 °C, changing the hydrogen pressure from 1 to 50 bar, in order to maximize the formation of 4a and 4c, respectively. The results are summarized in Figure 8a,b, respectively.

We were pleased to notice that at 25 °C the pressure affected both the conversion and the products’ distributions. Indeed, the increase of hydrogen pressure from 1 to 20 bar and 30 bar favored the conversion from 37% to 59% and >99%, respectively (Figure 8a). On the other side, the selectivity toward 4a decreased from >99 to 89% at 1 and 30 bar H_2_, respectively. Thereafter, no further changes were appreciated at 40 and 50 bar. A dramatically different behavior was observed at 100 °C for the reaction selectivity (Figure 8b). The distribution of 4a and 4b gradually decreased from 79% and 21% to 43% and 45% by increasing the H_2_ pressure from 1 to 50 bar, respectively. Overall, this trend reflected the progressively larger availability of H_2_ dissolved in the reaction mixture.

Nevertheless, the increase in the hydrogen pressure up to 50 bar did not favor the formation of the fully hydrogenated product 4c, which progressively increased from 3% up to 12% from 20 to 50 bar, respectively. In order to maximize the selectivity toward 4c, a further experiment was carried out by decreasing the flow rate from 0.3 mL min^−1^ up to 0.1 mL min^−1^, at 100 °C under 50 bar H_2_. Under such conditions, 4a, 4b and 4c were observed at 25%, 56% and 19%, respectively. In summary, parametric analysis showed that the catalytic performance of Pd-N/Ca in terms of selectivity toward 4a, 4b and 4c in the reductive amination of furfural with furfuryl amine could be influenced by both temperature and hydrogen pressure. By tuning the reaction parameters, 89% selectivity to 4a was reached at full conversion of 3, at 25 °C and 0.3 mL min^−1^, under 30 bar H_2_. Under these optimized reaction conditions, the stability of the catalyst over time was investigated.

### 3.4. Catalyst Stability

**Figure 9:**
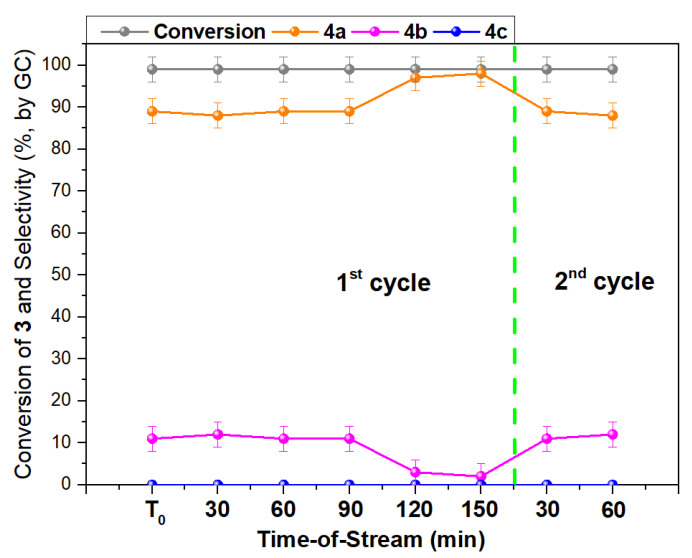
Catalyst stability. Reaction conditions: 3 (0.1 M in methanol), Pd-N/Ca (50 mg), 50 °C, 30 bar H_2_, flow rate (0.3 mL min^−1^). Conversion and selectivity were determined by GC.

A long-term stability analysis was eventually performed to demonstrate the stability of the catalytic system under the optimized reaction conditions of Figure 9, i.e., at 25 °C and 0.3 mL min^−1^, under 30 bar H_2_. Accordingly, the reaction was performed for 150 min time-on-stream. The results are summarized in Figure 9.

Despite the fact that almost no change in the reaction progress was noticed in the first 90 min, an increase of the selectivity to 4a (from 88–89% to 97–98%, respectively) was observed after 120 min, and a plateau was noticed up to 150 min of reaction, suggesting that a slight catalyst deactivation took place. A washing cycle was subsequently performed by pumping methanol in the continuous flow apparatus at 100 °C and 0.5 mL min^−1^ for 45 min, in order to check if the reduction of the activity was due to the adsorption of organic moieties on the active surface of the catalysts rather than deactivation. Pleasingly, the washing cycle restored the initial catalyst activity, most likely confirming that the slight drop in activity after 90 min could be due to the adsorbed compounds on the catalyst surface.

### 3.5. Reaction Pathway

**Figure 10:**
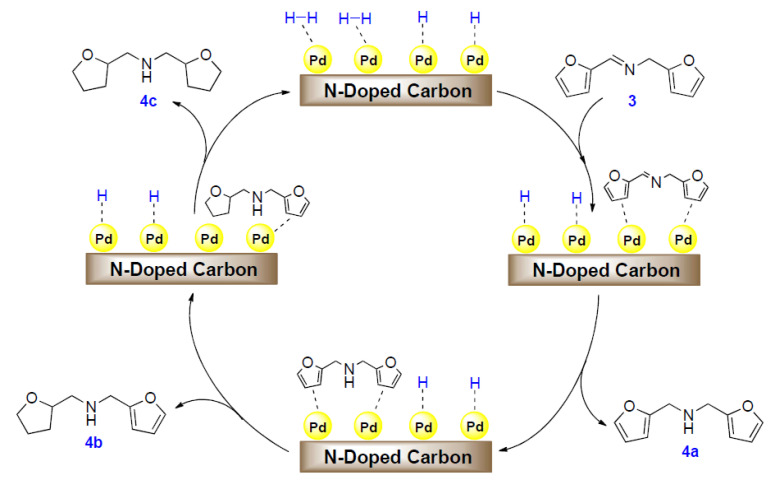
Proposed reaction pathway for the reductive amination of furfural with furfuryl amine over Pd-N/Ca system.

To explain the role of the Pd-N/Ca system in the reaction progress, the reaction pathway reported in Figure 10 hypothesizes that the reductive amination of furfural with furfuryl amine proceeds firstly with the adsorption of molecular hydrogen on Pd-sites, making the subsequent transfer of hydrogen on the C=N bond of 3 possible. The hydrogenation of 3 allows the formation of 4a, which could be further hydrogenated on the aromatic ring to 4b. The final hydrogenation of the furanic ring moiety of 4b leads to the formation of 4c.

## 4. Conclusions

In summary, through this contribution a novel strategy for the valorization of chitin was developed, employing this abundant biopolymer as a source of nitrogen and carbon for the preparation of *N*-doped carbonaceous materials. The aforementioned nitrogen-containing carbon-based samples were used as efficient supports for the incorporation of metal nanoparticles, specifically homogeneously distributed and well-dispersed Pd entities, which, according to the deposition method, displayed different dimensions. The materials were characterized and compared following a multi-technique approach and insights into their catalytic activity were attained in the reductive amination reaction of furfural with furfuryl amine, also promoting strategies for the valorization of biomass-derived platform molecules. Remarkably, the reaction was carried out under continuous flow conditions and a complete parametric analysis was performed with the ability to tune the products’ selectivity by controlling the reaction temperature and pressure. This work represents an example of the potentialities of biomass-derived materials—in particular chitin as a carbon and nitrogen source—and could inspire the scientific community, not only with regard to the use of chitin, but also with regard to the use of chitin containing-biomass wastes (e.g., shrimp shells, crab shells) for the preparation of sustainable advanced nanomaterials.

## Figures and Tables

**Table 1:** Textural properties of the metal-supported *N*-doped carbonaceous materials.

Material	*S*_BET_ (m^2^/g) ^a^	*D*_BJH_ (nm) ^b^	*V*_BJH_ (cm^3^/g) ^c^	Pd Concentration/mg g^−1 d^	Pd Concentration/wt.% ^e^
N/C	320	3.9	0.35	N.D. ^f^	N.D. ^f^
Pd-N/C_a_	300	4.3	0.25	7.1	6.0
Pd-N/C_b_	311	4.0	0.28	6.9	5.9

^a^*S*_BET_: Specific surface area was calculated by the Brunauer–Emmett–Teller (BET) equation. ^b^*D*_BJH_: Mean pore size diameter was calculated by the Barret–Joyner–Halenda (BJH) equation. ^c^*V*_BJH_: Pore volumes were calculated by the Barret–Joyner–Halenda (BJH) equation. Pd concentrations were determined by ^d^ ICP-OES and ^e^ EDX analyses. ^f^ N.D.: denotes not detected.

## Data Availability

Not applicable.

## Supplementary Materials

The following supporting information can be downloaded at: https://www.mdpi.com/article/10.3390/ma16020575/s1, Figure S1: Mass Spectrum of **3** (EI, 70 eV). Figure S2. Mass Spectrum of **4a** (EI, 70 eV). Figure S3. Mass Spectrum of **4b** (EI, 70 eV). Figure S4. Mass Spectrum of **4c** (EI, 70 eV).
