# Supplementary material for: Chitin-Derived Nanocatalysts for Reductive Amination Reactions
Source: Materials (Basel). 2023 Jan 6;16(2):575. doi: 10.3390/ma16020575 (PMC9864054; doi:10.3390/ma16020575)
Supplement: Supplementary file 1 [file materials-16-00575-s001.zip › materials-2130267-supplementary.pdf]

SUPPORTING INFORMATION

Chitin-Derived Nanocatalysts for Reductive Amination Reactions

Daniele Polidoro [1], Daily Rodriguez-Padron [1*], Alvise Perosa [1], Rafael Luque [2,3] and Maurizio Selva [1,*]

[1] Department of Molecular Science and Nanosystems, Ca' Foscari University of Venice, Via Torino 155, 30175 Venezia Mestre, Italy

[2]Departament of Natural Sciences, Mid Sweden University, Holmgatan 10, 85170 Sundsvall, Sweeden.

[3] Universidad ECOTEC, Km. 13.5 Samborondón, Samborondón, EC092302, Ecuador

* D.R.-P. daily.rodriguez@unive.it; *M.S. selva@unive.it

Charcterization data

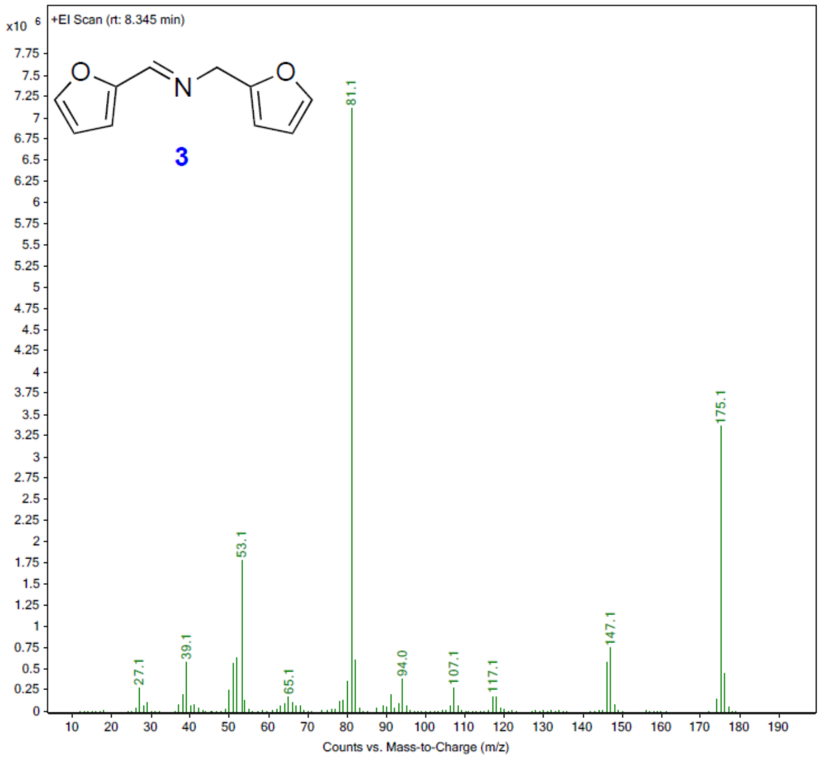

Figure S1. Mass Spectrum of **3** (EI, 70 eV).

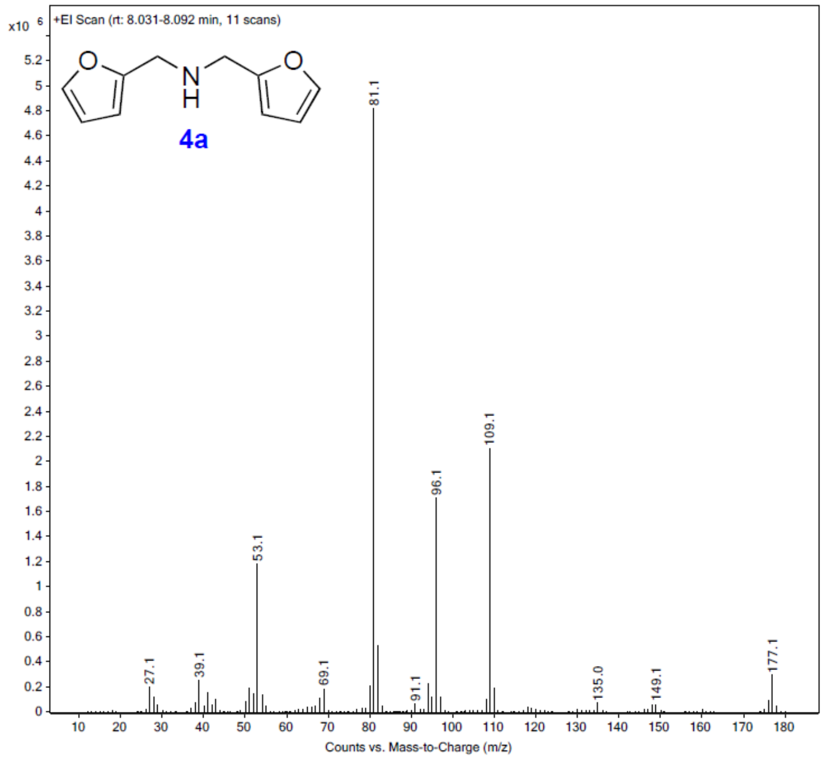

Figure S2. Mass Spectrum of **4a** (EI, 70 eV).

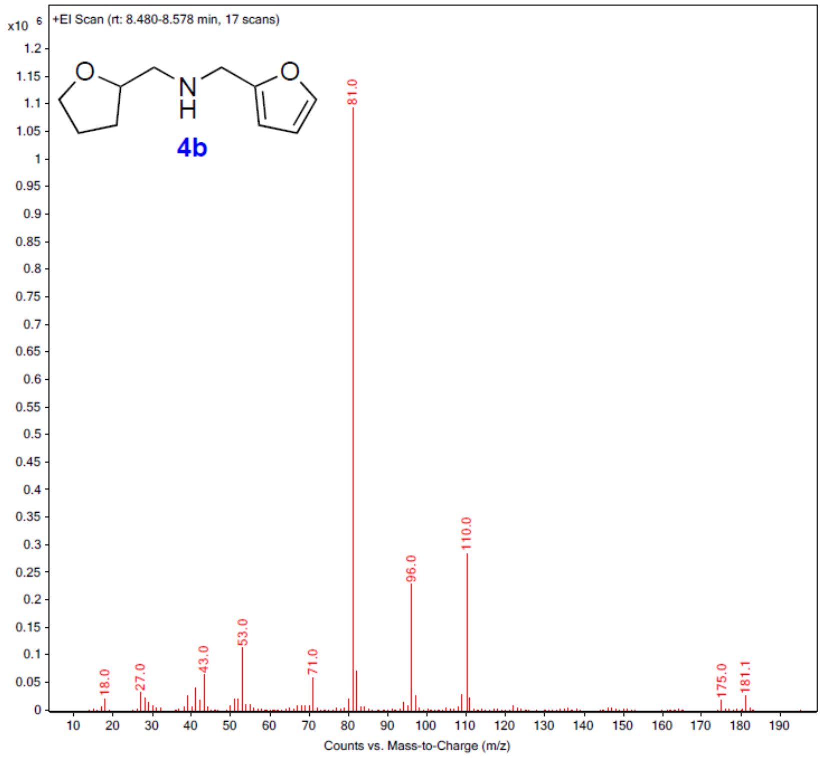

Figure S3. Mass Spectrum of **4b** (EI, 70 eV).

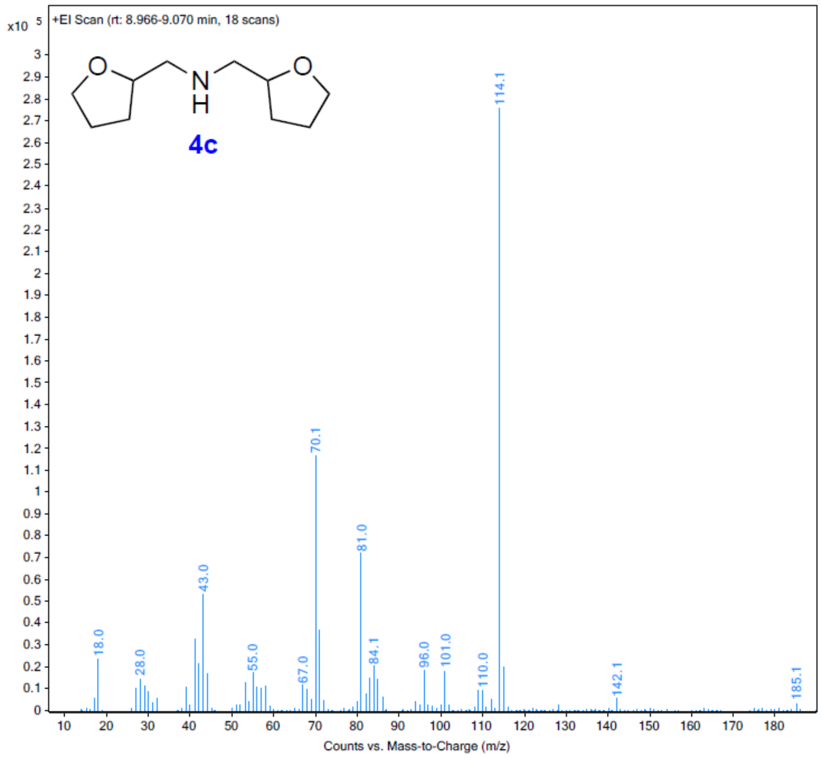

Figure S4. Mass Spectrum of **4c** (EI, 70 eV).
